# Rising Trends in Medication Non-compliance and Associated Worsening Cardiovascular and Cerebrovascular Outcomes Among Hospitalized Adults Across the United States

**DOI:** 10.7759/cureus.5389

**Published:** 2019-08-14

**Authors:** Rupak Desai, Samarthkumar Thakkar, Hee Kong Fong, Yash Varma, Mir Z Ali Khan, Vikram B Itare, Jilmil S Raina, Sejal Savani, Nanush Damarlapally, Rajkumar P Doshi, Kishorbhai Gangani, Kranthi Sitammagari

**Affiliations:** 1 Cardiology, Atlanta Veterans Affairs Medical Center, Decatur, USA; 2 Internal Medicine, Rochester General Hospital, Rochester, USA; 3 Cardiovascular Medicine, University of California Davis Medical Center, Sacramento, USA; 4 Internal Medicine, Government Medical College, Bhavnagar, IND; 5 Internal Medicine, Saint Peters University Hospital, New Brunswick, USA; 6 Internal Medicine, Smolensk State Medical University, Smolensk, RUS; 7 Public Health, New York University, New York, USA; 8 Health Sciences, Coleman College of Health Sciences, Houston, USA; 9 Internal Medicine, University of Nevada, Reno School of Medicine, Reno, USA; 10 Internal Medicine, Texas Health Arlington Memorial Hospital, Arlington, USA; 11 Internal Medicine, Atrium Health Union, Monroe, USA

**Keywords:** medication non-compliance, non adherence, cardiovascular diseases, cerebrovascular disease, stroke, myocardial infarction, arrhythmias, venous thromboembolism, trends, mortality

## Abstract

Introduction

Small-scale studies have described concerning rates of non-compliance/nonadherence towards groups of medications for primary and secondary prevention. Trends in cardiovascular and cerebrovascular events (CCE) among hospitalized patients with a non-compliant behavior towards medication, on the whole, remains unexplored on a large scale.

Methods

Using the National Inpatient Sample databases (2007-2014), we sought to assess the prevalence and trends in all-cause mortality and CCE in adult patients hospitalized with medication non-compliance. We compared baseline characteristics and comorbidities in the non-compliant patients with and without concomitant in-hospital CCE.

Results

We identified 7,453,831 adult hospitalizations with medication non-compliance from 2007 to 2014, of which 867,997 (11.6%) patients demonstrated in-hospital CCE. Non-compliant patients with CCE consisted of a higher number of older, white, male patients having greater comorbid risk factors. Non-compliant patients with CCE had higher all-cause in-hospital mortality (3% vs. 0.7%), frequent transfers [4.4% vs. 1.8% transfers to short-term hospitals, and 17.6% vs. 11.6% other transfers (skilled nursing or intermediate care facilities)], lower routine discharges (59.4% vs. 71.1%), and higher mean hospital charges ($52,740 vs. $30,748) compared to non-compliant patients without CCE. Remarkably, this study demonstrates the rising trend in medication non-compliance across all age, sex, and race groups, and related in-hospital mortality, CCE, transfers to other facilities, and the health care cost from 2007 to 2014.

Conclusions

We observed rising trends in the prevalence of medication non-compliance and subsequent in-hospital mortality in hospitalizations among adults from 2007 to 2014. Non-compliant patients with inpatient CCE demonstrated rising trends in all-cause mortality, complications, health care utilization, and cost from 2007 to 2014.

## Introduction

According to the World Health Organization, medication non-compliance or nonadherence has emerged as a major problem in developing countries where only 50% adherence has been found among patients with a long-term illness and the problem is expected to be greater in underdeveloped countries in view of the prevailing dearth of health care resources and disparities in health care delivery [1]. A meta-analysis of 376,162 patients established the positive effect of guideline-directed medical therapy (GDMT) for both the primary and secondary prevention of cardiovascular disease (CVD) and concluded that measures to improve overall adherence are desirable as opposed to class-specific adherence [2]. A meta-analysis of 21 studies revealed that GDMT can reduce the risk of all-cause mortality by 40% and cardiovascular events by 30% in patients with established CVD [3]. Similarly, a dose-response analysis discovered that higher compliance with antihypertensive medication was associated with a lower risk of both hemorrhagic and ischemic stroke [4]. Therefore, nonadherence to antihypertensive medications has shown to increase both cardiovascular and cerebrovascular events (CCE) and related mortality [5]. Multiple factors can contribute towards poor adherence which can be divided into socioeconomic, motivational, and communicational causes [6]. Earlier studies have evaluated the outcomes in the form of mortality and cost of care related to non-compliance to a group of medications mainly including antihypertensive, antidiabetics, or lipid-lowering agents [7-9]. However, studies describing the sequential intersection of multiple sociodemographic and comorbid variables influencing medication non-compliance and cardiovascular outcomes would be crucial to perform as prior studies have called for improvement in overall patient adherence/compliance rather than just towards a class of medication [2]. Hence, using a nationally representative cohort from the National Inpatient Sample (NIS) databases (2007-2014), we postulate to reveal the rising prevalence and concerning trends in medication non-compliance, all-cause inpatient mortality, and CCE among patients with non-compliant behaviors irrespective of class of medications. We also compared characteristics and manifest comorbidities in non-compliant patients with and without incidental CCE. 

## Materials and methods

Data source

This retrospective study analyzed the NIS databases (2007-2014), which is a division of the Healthcare Cost and Utilization Project, developed by the Agency for Healthcare Research and Quality. The NIS is the largest publicly available hospital discharge administrative database consisting of 20% stratified sampling of all inpatient admissions (more than seven million hospital stays per year) to non-federal hospitals in the United States. Considering the de-identified nature of the admission records, an approval from the institutional review board was not mandatory for this study.

Study population and variables

From January 2007 to December 2014, adult patients were identified with a non-compliant behavior towards medications using the International Classification of Diseases, Ninth Revision, Clinical Modification diagnostic code V15.81 [10]. These patients were divided into two study groups: those who experienced CCE vs. those who did not. We evaluated the frequency of various CCE which included first or subsequent myocardial infarction (MI), first or subsequent stroke, out of hospital cardiac arrest, arrhythmias, and venous thromboembolism (Appendix). The details of NIS data on variables, demographics, hospital-level characteristics, and comorbidities have been published previously [11-13]. We examined and compared the following baseline variables between the two groups: demographics, the source of payer and median household income quartile for patients’ zip code, hospital-related variables, and pre-existing comorbidities.

Primary and secondary outcomes

The primary outcomes were defined as in-hospital mortality, disposition, and health care resource utilization in terms of mean length of stay (LOS, days) and hospital charges (USD). The secondary outcomes were the trends in all-cause in-hospital mortality and CCE in patients admitted with medication nonadherence regardless of medication group from 2007 to 2014.

Statistical analyses

Statistical analyses were performed using SPSS V.22.0 (IBM Corp., Armonk, NY, USA). Pearson’s chi-square test is used for the categorical variables which were presented as numbers and proportions, and the Student t-test was used for the continuous variables which were presented as the mean and standard deviation. Furthermore, we stratified the population into the age groups of 18-44, 45-64, and ≥65 years. A two-sided p-value ≤0.05 was defined as a statistical significance.

## Results

From 2007 to 2014, we identified a total of 7,453,831 hospitalizations among the adult population with a history of medication nonadherence. Of these, 867,997 (11.6%) patients experienced in-hospital CCE. Table 1 displays the patients and hospital-related characteristics as well as pre-existing comorbidities in nonadherent adults with and without CCE. Patients non-compliant to medications who experienced CCE consisted of higher number of older (mean age 59.6 vs. 51.0 years, 45-64 years: 50.1% vs. 43.8%), white (58.0% vs. 50.5%), male (62.1% vs. 37.9%) patients. The CCE cohort more often consisted of Medicare enrollees (44.5% vs. 39.6%) and had emergency admissions (95.1% vs. 91.9%). The Southern and West regions documented a higher number of CCE among non-compliant patients. The incidence of major comorbid risk factors such as diabetes, hypertension, obesity and dyslipidemia, peripheral vascular disease, smoking, chronic pulmonary disease, coagulopathy, renal failure, as well as valvular heart disease was also higher in patients with CCE.

**Table 1:** Baseline Characteristics of Adult Patients Hospitalized with Medication Non-compliance with vs. Without In-hospital Cardiovascular/Cerebrovascular Events P-values <0.05 indicate statistical significance. ^†^HMO=Health Maintenance Organization. CCE=cardiovascular and cerebrovascular events. Cardiovascular and cerebrovascular events included first or subsequent myocardial infarction, first or subsequent stroke, out-of-hospital cardiac arrest, arrhythmias, and venous thromboembolism.

Variable	Hospitalizations with Medication Non-compliance (n=7,453,831)		P
	No CCE (n=6,585,834)	Yes CCE (n=867,997)	
Age (years) at hospitalization			
Mean age ± SD	51.0±17.0	59.6±14.7	<0.001
18-44	34.7%	14.3%	
45-64	43.8%	50.1%	
≥65	21.5%	35.6%	
Male	55.4%	62.1%	<0.001
Race			<0.001
White	50.5%	58.0%	
African American	33.1%	27.0%	
Hispanic	10.9%	9.3%	
Asian or Pacific Islander	1.7%	2.2%	
Native American	0.8%	0.6%	
Other	3.0%	2.9%	
Primary Expected Payer			<0.001
Medicare	39.6%	44.5%	
Medicaid	27.5%	17.2%	
Private including HMO†	16.6%	21.2%	
Self – pay/no charge/others	16.3%	17.2%	
Median Household Income			<0.001
0-25th	40.6%	36.9%	
26-50th	25.2%	25.9%	
51-75th	20.1%	21.7%	
76-100th	14.1%	15.5%	
Non-elective admission	91.9%	95.1%	<0.001
Bed size of hospital			<0.001
Small	12.5%	11.2%	
Medium	26.2%	25.1%	
Large	61.3%	63.7%	
Location/Teaching Status of Hospital			<0.001
Rural	8.9%	8.3%	
Urban - non-teaching	38.2%	39.2%	
Urban - teaching	52.9%	52.5%	
Region of Hospital			<0.001
Northeast	20.4%	16.7%	
Midwest	22.8%	22.1%	
South	40.0%	43.3%	
West	16.8%	17.9%	
Comorbidities			
Alcohol abuse	14.1%	11.5%	<0.001
Deficiency anemias	19.5%	16.6%	<0.001
Congestive heart failure	9.2%	8.3%	<0.001
Dyslipidemia	26.6%	47.8%	<0.001
Smoking	41.1%	46.6%	<0.001
Chronic pulmonary disease	21.8%	22.6%	<0.001
Coagulopathy	4.4%	4.9%	<0.001
Depression	11.8%	10.2%	<0.001
Diabetes, uncomplicated	22.7%	31.4%	<0.001
Diabetes with chronic complications	8.3%	8.4%	0.174
Drug abuse	18.1%	9.8%	<0.001
Hypertension	50.9%	73.7%	<0.001
Hypothyroidism	8.4%	8.4%	0.119
Liver disease	4.9%	3.0%	<0.001
Fluid and electrolyte disorders	27.7%	25.7%	<0.001
Obesity	16.7%	19.9%	<0.001
Peripheral vascular disorders	5.5%	8.8%	<0.001
Pulmonary circulation disorders	1.9%	4.4%	<0.001
Renal failure	14.8%	17.6%	<0.001
Valvular heart disease	2.6%	3.5%	<0.001

**Figure 1:**
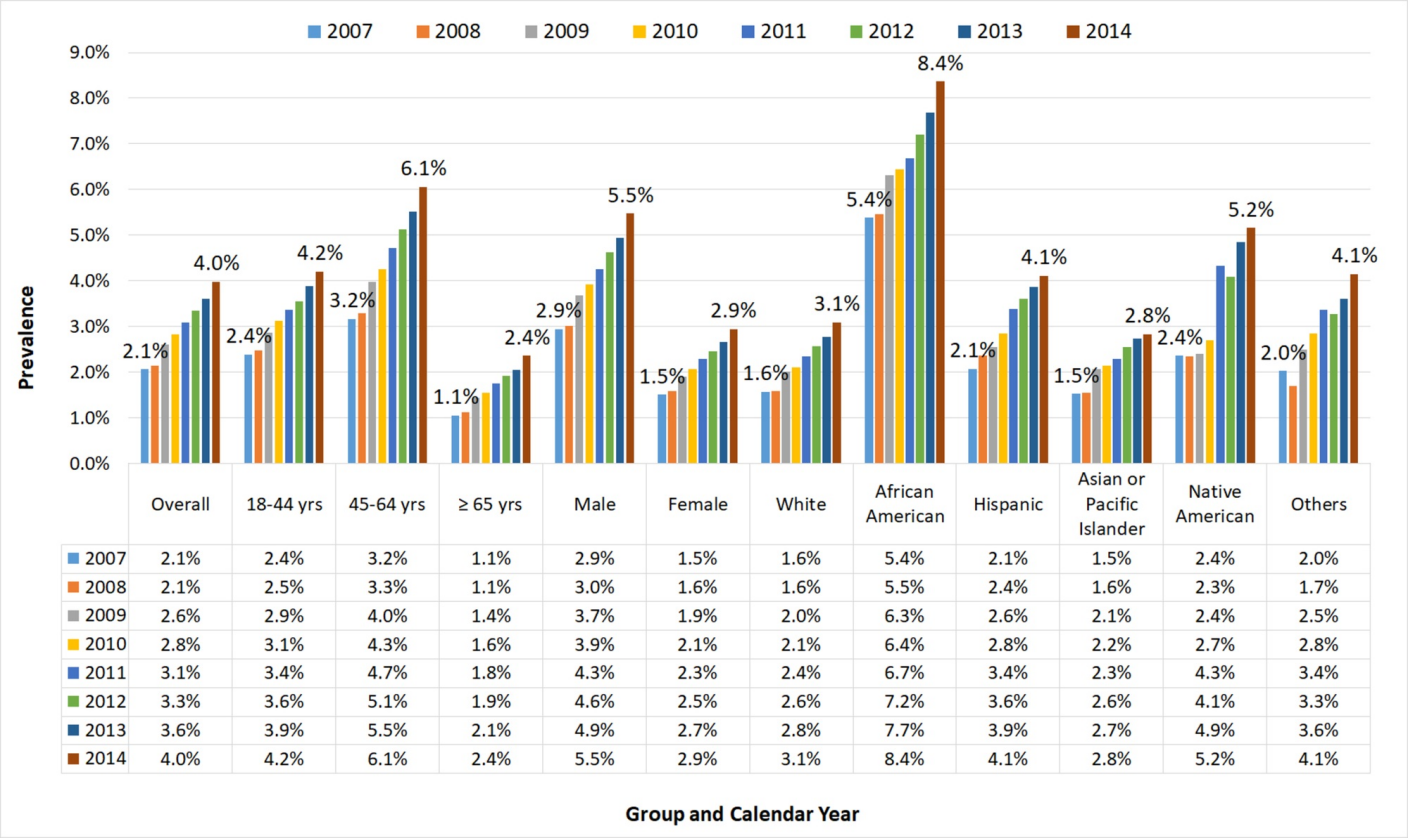
Trends in Prevalence of Medication Non-compliance Stratified by Age, Sex, and Race Among Adult Patients Hospitalized from 2007 to 2014

Figure 1 demonstrates time trends in the prevalence of medication non-compliance among adult patients hospitalized from 2007 to 2014 according to their age group, gender, and race. The study demonstrates a rising trend in each age group, each race of people, and both the genders. Among the age groups, 45-64 years age group had the highest prevalence of medication non-compliance (3.2% in 2007 to 6.1% in 2014) followed by 18-44 years and ≥65 years age groups. Similarly, based on the gender, males (2.9% in 2007 to 5.5% in 2014) and among the race groups, African Americans (5.4% in 2007 to 8.4% in 2014) had the highest prevalence of medication nonadherence followed by Native American, Hispanic, others, White, and Asian or Pacific Islander. 

**Figure 2:**
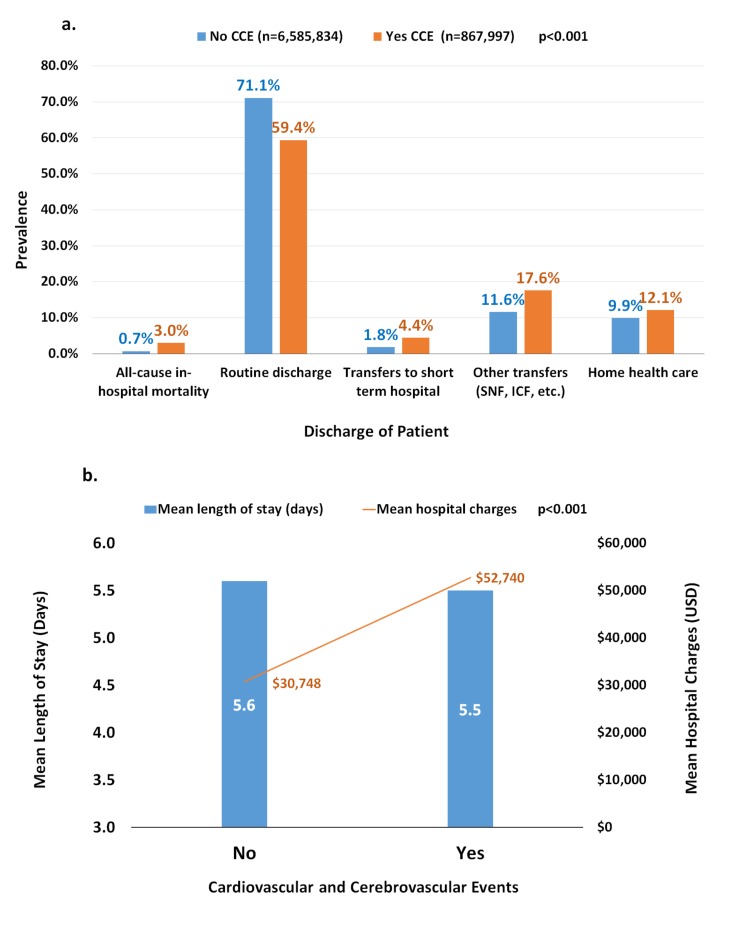
In-hospital Outcomes and Health Care Resource Utilization in Non-compliant Adult Patients with vs. Without In-hospital Cardiovascular and Cerebrovascular Events a. In-hospital outcomes and disposition in non-compliant patients with vs. without in-hospital cardiovascular and cerebrovascular events. b. Mean length of stay (days) and hospital charges (United States dollar) in non-compliant patients with vs. without in-hospital cardiovascular and cerebrovascular events. CCE=cardiovascular and cerebrovascular events. Cardiovascular or cerebrovascular events included first or subsequent myocardial infarction, first or subsequent stroke, out-of-hospital cardiac arrest, arrhythmias, and venous thromboembolism.

Figure 2 demonstrates in-hospital outcomes as well as mean LOS and hospital charges with or without CCE in patients with medication non-compliance. All-cause in-hospital mortality was significantly higher in patients with CCE compared to without such events (3% vs. 0.7%). In terms of disposition, the non-CCE group had a significantly lower number of routine discharges than patients with CCE (59.4% vs. 71.1%), whereas a greater number of transfers were associated with the CCE group (4.4% vs. 1.8% transfers to short-term hospitals and 17.6% vs. 11.6% other transfers including skilled nursing facility, intermediate care facility, etc.). Though the mean LOS was equivalent in both the groups, mean hospital charges were considerably higher in non-compliant patients with CCE vs. without CCE ($52,740 vs. $30,748). 

Table 2 demonstrates time trends for in-hospital mortality, cardiovascular and cerebrovascular complications, disposition, mean LOS, as well as hospital charges in hospitalized patients with a history of medication non-compliance from 2007 to 2014. The study demonstrated a rising trend of all-cause in-hospital mortality from 0.7% in 2007 to 1.1% in 2014. Similarly, over time, prevalence of many CCE also displayed an upward sloping trend whether it was 2% of first MI in 2007 to 2.5% in 2014, 2.9% of the first stroke and 0.1% of consecutive stroke in 2007 to 3% and 0.6% in 2014, respectively. Similarly, the proportion of venous thrombosis, out-of-hospital cardiac arrest, and arrhythmia were also higher in 2014 compared to 2007. However, the proportion of patients discharged routinely and transferred to short-term hospital decreased consistently from 2007 to 2014 (62.2% in 2007 to 56.9% in 2014). But the trend for transfers to other facilities (skilled nursing facility, intermediate care facility, etc) and home health care increased since 2007. Though inconsistently, the mean LOS rose from 5.17 days in 2007 to 5.69 days in 2014 and finally the health care cost also consistently increased from $36,423 in 2007 to $62,341 in 2014. (For all the aforementioned results, p-value was <0.001).

**Table 2:** Trends in the In-hospital Mortality, Complications, and Health Care Resource Utilization in Hospitalized Adults with Medication Non-compliance P_trend_ value <0.05 indicates statistical significance. ^†^SNF=skilled nursing facility, ^‡^ICF=intermediate care facility. ↑ indicates rising trends and ↓ indicates declining trends.

Outcomes	Overall	2007	2008	2009	2010	2011	2012	2013	2014	P_trend_	Direction
All-cause in-hospital mortality	1.0%	0.7%	0.8%	1.0%	0.9%	1.0%	1.0%	1.0%	1.1%	<0.001	↑
First myocardial infarction	2.3%	2.0%	2.2%	2.3%	2.2%	2.3%	2.4%	2.4%	2.5%	<0.001	↑
First stroke	2.9%	2.9%	2.8%	2.6%	2.9%	2.9%	2.9%	3.0%	3.0%	<0.001	↑
Subsequent myocardial infarction	1.5%	1.2%	1.3%	1.4%	1.4%	1.6%	1.6%	1.6%	1.6%	<0.001	↑
Subsequent stroke	0.4%	0.1%	0.3%	0.4%	0.5%	0.5%	0.5%	0.6%	0.6%	<0.001	↑
Venous thromboembolic events	2.2%	1.9%	2.0%	2.2%	2.1%	2.3%	2.3%	2.3%	2.5%	<0.001	↑
Out-of-hospital cardiac arrest	0.21%	0.17%	0.17%	0.20%	0.21%	0.22%	0.23%	0.21%	0.25%	<0.001	↑
Arrhythmia	17.4%	13.6%	14.7%	16.3%	16.7%	18.1%	18.5%	18.6%	20.0%	<0.001	↑
Disposition											
Routine discharge	59.4%	62.2%	63.3%	60.6%	59.3%	58.4%	59.5%	58.6%	56.9%	<0.001	↓
Transfers to short-term hospital	4.4%	5.5%	4.6%	4.4%	4.8%	4.6%	4.0%	3.8%	4.0%	<0.001	↓
Other transfers (SNF^†^, ICF^‡^, etc.)	17.6%	15.6%	15.3%	16.9%	17.6%	18.3%	17.5%	18.3%	19.4%	<0.001	↑
Home health care	12.1%	10.2%	10.4%	11.2%	12.0%	12.6%	12.8%	12.8%	13.1%	<0.001	↑
Mean length of stay (days)	5.52	5.17	5.13	5.63	5.64	5.56	5.48	5.56	5.69	<0.001	↑
Mean hospital charges	$52,740	$36,423	$39,437	$48,278	$50,460	$54,274	$55,643	$59,701	$62,341	<0.001	↑

## Discussion

Our findings obtained in a study of a large nationwide cohort indicated that nearly 12% of patients hospitalized with a history of medication non-compliance developed CCE and had suffered higher in-hospital mortality, upward trends in all-cause mortality and in-hospital cardiovascular complications, and higher resource utilization as compared to non-compliant patients without CCE. Patients developing CCE had a lower rate of routine discharge and a higher rate of transfers to other facilities and requirement of home health care. The health care cost was also significantly higher in non-compliant patients with CCE. Our study showed increasing trends in the in-hospital mortality and complications related to CCE from 2007 to 2014. 

Adherence is a challenge mainly for chronic diseases, especially during the symptom-free intervals. The consequences of nonadherence are disease mismanagement, progression, and development of complications which lead to increased use of medical resources in the form of frequent health care visits, dependence on health care facilities and hospitalizations, and all these lead to poor outcomes in terms of quality of life, increased mortality, and greater cost of care [14]. In the United States, excess hospitalizations due to medication nonadherence cost approximately $100 billion every year [15]. Several studies have found that overall health care costs are much higher for patients with poor adherence as compared to patients with good adherence. A review summarizing the impact of nonadherence on health care cost in several chronic diseases such as diabetes, CVD, and chronic obstructive pulmonary disease concluded that increasing the medication adherence significantly reduces the overall health care cost [16]. Our prior analyses have revealed close etiopathological and prognostic link of cardiovascular disorders and outcomes with endocrine, respiratory, or psychiatric disorders which also suggests that medications targeted for one organ system can help prevent overall complications, improve quality of life, and improve long-term prognosis [13, 17-21].

Correspondingly, several studies have described how nonadherence increases individual comorbid diseases or risk factors and in turn, in-hospital mortality. One study by Rasmussen et al. explored the association between drug adherence and mortality in survivors of acute MI which showed that in comparison with the patients with high levels of adherence to statins, the risk of mortality was 25% higher among patients with poor adherence [22]. Similarly, one study assessing the risk of fatal stroke associated with nonadherence to statin and antihypertensive therapy derived that individuals with hypercholesterolemia and hypertension who failed to take their prescribed medications experienced a substantially increased (up to twice) risk of fatal stroke [23]. Studies have established the demographics of patients more frequently found to be linked to medication non-compliance. As one leap forward, we studied the baseline characteristics and comorbidities among non-compliant patients with CCE and compared it to patients without CCE to help clinicians to understand the demographics of the nonadherent high-risk patient population. We observed that the patients with CCE were older, more frequently white, men and had a higher prevalence of comorbidities. Non-compliant patients developing CCE had worse outcomes, underwent excessive health care utilization (transfers to short-term hospitals, intermediate care, and skilled nursing facilities, home health care) and in turn, the higher cost of care per admission. Our findings from a much larger patient population linking the medication nonadherence to in-hospital outcomes expand the literature on nonadherence and emphasize the importance of medication adherence in clinical practice.

The challenge with medication nonadherence is that there does not exist a particular parameter to measure it and it is invisible to patients, families, and health care providers. To deal with the emerging concerns pertinent to nonadherence, we should identify the barriers related to nonadherence which come under the categories of patient-related factors which include poor knowledge (most common predictor), lack of motivation, depression, denial, and cultural beliefs; treatment-related factors which include the type of illness, complexity of treatment, side effects, cost, and others most importantly physician-patient relationship [24, 25]. Then comes the methods of measuring nonadherence such as directly observed therapy, identifying the level of medicine in the blood (most reliable); indirect methods such as patient questionnaires, pill counts, clinical response, electronic monitors, patient diary, and many more [26]. Even after incorporating different interventions to increase the drug adherence including prescribing generic medications, simplifying the schedule, patients’ health coaching, close follow-up, frequent communication with treating physicians, and using mobile health care applications [26-29], there are number of population-based studies showing the decreasing compliance rate for multiple medications in the short term and long term [30]. The trends in hospitalizations with worsened outcomes heightened complications, and health care costs related to medication nonadherence were consistently evident from our study results.

Limitations

This study has several limitations. The current analysis does not reveal nonadherence to a specific group of medication or disease. Because of the retrospective nature of the study, it represents only an association and not causation. Over- or underreporting of the study population could be likely secondary to administrative coding errors in the NIS database. Despite a few limitations, a large sample size increases the generalizability of the study findings.

## Conclusions

In this population-based nationwide analysis, we observed concerning trends in the prevalence of medication non-compliance and related CCE among hospitalized adults from 2007 to 2014 which was associated with higher in-hospital mortality, health care utilization, and cost. This study highlights the role of health care practitioners in screening older patients with chronic conditions for poor medication compliance and incorporating a more robust and collaborative approach which may possibly make a difference in clinical medicine on a global level. 

## Appendices

**Table 3:** International Classification of Diseases, 9th Revision, Clinical Modification (ICD-9-CM)/Clinical Classifications Software (CCS) Codes Used to Identify Cardiovascular Events and Outcomes CCS is based on the International Classification of Diseases, 9th Revision, Clinical Modification (ICD-9-CM), and a uniform and standardized coding system. The ICD-9-CM's multitude of codes - over 14,000 diagnosis codes and 3,900 procedure codes - are collapsed into a smaller number of clinically meaningful categories that are sometimes more useful for presenting descriptive statistics than are individual ICD-9-CM codes. Source: HCUP CCS. Healthcare Cost and Utilization Project (HCUP). March 2017. Agency for Healthcare Research and Quality, Rockville, MD. www.hcup-us.ahrq.gov/toolssoftware/ccs/ccs.jsp. Accessed July 22, 2019.

Disease/Event/Outcome	ICD-9 CM/CCS Code
Acute myocardial infarction	CCS 100
Arrhythmia	CCS 106
Acute cerebrovascular disease/stroke	CCS 109
Previous myocardial infarction	ICD-9 412
Previous transient ischemic attack/stroke	ICD-9 V12.54
Venous thromboembolism	ICD-9 415.1x, 451.1x, 451.2, 451.8x, 451.9, 453.2, 453.4x, 453.8x, 453.9
Subsequent myocardial infarction	Primary discharge diagnosis of acute myocardial infarction (CCS 100) with any of secondary discharge diagnoses of previous myocardial infarction (ICD-9 412)
Subsequent stroke	Primary discharge diagnosis of acute cerebrovascular disease (CCS 109) with any of secondary discharge diagnoses of previous transient ischemic attack/stroke (ICD-9 V12.54)
Percutaneous coronary intervention	CCS 45
Coronary artery bypass grafting	CCS 44
